# Typologies in GPs’ referral practice

**DOI:** 10.1186/s12875-016-0495-y

**Published:** 2016-07-18

**Authors:** Olav Thorsen, Miriam Hartveit, Jan Olav Johannessen, Lars Fosse, Geir Egil Eide, Jörn Schulz, Anders Bærheim

**Affiliations:** Department of Global Public Health and Primary Care, University of Bergen, Box 7800, Bergen, N-5020 Norway; Department of Research, Stavanger University Hospital, Box 8100, Stavanger, N-4068 Norway; Department of Orthopaedics, Stavanger University Hospital, Box 8100, Stavanger, N-4068 Norway; Centre for Clinical Psychosis Research, Division of Psychiatry, Stavanger University Hospital, Box 8100, Stavanger, N-4068 Norway; Faculty of Social Sciences, University of Stavanger, Box 8100, Stavanger, N-4068 Norway; Section for Research and Innovation, Helse Fonna HF, Box 2170, Haugesund, N-5504 Norway; Centre for Clinical Research, Haukeland University Hospital, Box 1400, Bergen, N-5021 Norway; Section of Biostatistics, Stavanger University Hospital, Box 8100, Stavanger, N-4068 Norway

**Keywords:** Referral process, Typologies, Confidence, Uncertainty

## Abstract

**Background:**

GPs’ individual decisions to refer and the various ways of working when they refer are important determinants of secondary care use. The objective of this study was to explore and describe potential characteristics of GPs’ referral practice by investigating their opinions about referring and their self-reported experiences of what they do when they refer.

**Methods:**

Observational cross-sectional study using data from 128 Norwegian GPs who filled in a questionnaire with statements on how they regarded the referral process, and who were invited to collect data when they actually referred to hospital during one month. Only elective referrals were recorded. The 57 participants (44,5 %) recorded data from 691 referrals. The variables were included in a principal component analysis. A multiple linear regression analysis was conducted to identify typologies with GP’s age, gender, specialty in family medicine and location as independent variables.

**Results:**

Eight principal components describe the different ways GPs think and work when they refer. Two typologies summarize these components: *confidence* characterizing specialists in family medicine, mainly female, who reported a more patient-centred practice making priority decisions when they refer, who confer easily with hospital consultants and who complete the referrals during the consultation; *uncertainty* characterizing young, mainly male non-specialists in family medicine, experiencing patients’ pressure to be referred, heavy workload, having reluctance to cooperate with the patient and reporting sparse contact with hospital colleagues.

**Conclusions:**

Training specialists in family medicine in patient-centred method, easy conference with hospital consultant and cooperation with patients while making the referral may foster both self-reflections on own competences and increased levels of confidence.

**Electronic supplementary material:**

The online version of this article (doi:10.1186/s12875-016-0495-y) contains supplementary material, which is available to authorized users.

## Background

In many countries there is a long tradition for general practitioners to take care of most health problems, leaving the hospital specialists to do the things that they only can perform [1]. In Norway all residents are connected to a regular GP. All inpatient treatment is free. The gatekeeping system means that patients need a referral from their GP to be examined or treated in specialist health services. Except for urgent cases, such as accidents or emergency situations, the decision to refer is the start of the patient’s clinical course into specialist care.

In many countries referral rates have increased dramatically during the last decades [2, 3] and the consequences for the society are more use of specialist health care and greater expenses [2, 4–7]. There are many reasons for this trend, such as better access to specialist services, cultural changes, national laws and regulations and patients' requirements [8, 9]. The GPs’ individual decisions to refer vary greatly and cannot be explained by patient morbidity alone [10–12]. In 2011we showed that GPs regarded the referring process as asymmetric and sometimes embarrassing and wanted improved dialogue with hospital specialists [13]. GPs are often in a squeezed position between a patient with a demand for a referral to a hospital specialist and the unease felt when sending an inappropriate or unnecessary referral letter. Hospital consultants request better communication, like a telephone call before referring. Many referrals are regarded as unnecessary, meaning that the problem could be handled by the GP [8, 14]. Improving the quality of the referral process is important to facilitate timely access to specialty care [15–17]. Studies have shown that better e-communication between GPs and hospital consultants and more advanced electronic referral decision may facilitate this process [18, 19]. Continuous professional development (CPD) groups with certified supervisors, where the participants discuss clinical problems and difficulties in the consultation room can help young GPs to become more confident and safe in their role as a GP and specialist in family practice [20]. More knowledge is needed on the reasons for GPs’ varying referral behaviours. The aim for this study was to explore and describe potential characteristics of GPs’ referral practice by investigating their opinions about referring and their self-reported experiences of what they do when they refer.

## Methods

### Study design and participants

**Fig. 1:**
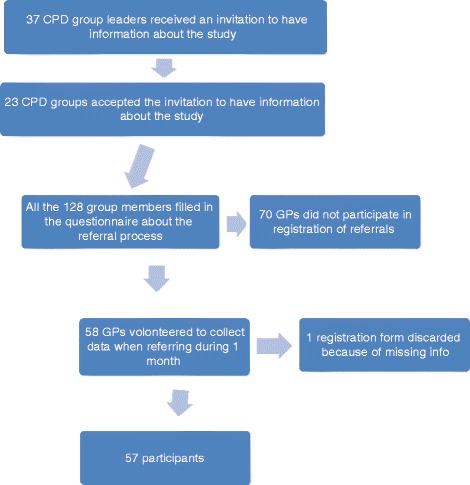
Flowchart study participants

We did an observational cross-sectional study on GPs’ attitudes to and perceptions about their usual referral process and on what they actually did when they sent elective referrals to hospital for admission or outpatient opinion. As no identical studies had been done before, we designed the questionnaire (Additional file 1: Appendix 1) and the referral registration form (Additional file 1: Appendix 2) on the basis of the results from a previous study [13] in collaboration with experienced academic and non-academic GPs. We piloted the questionnaire and referral registration form in another CPD group outside the present research area, without having any suggestions for changes. In December 2013 we sent information about the study to the group leaders of all the 37 CPD groups, (around 250 GPs) in the southern part of Rogaland County in Norway, a region with 330 000 inhabitants, 300 GPs and one hospital (Stavanger University Hospital). Of these, 23 groups accepted the invitation to have a meeting about the study. The meetings were held from January to April 2014. The 128 CPD group members were informed about the study and were asked to fill in a questionnaire about the referral process. They were then invited to collect data when referring to hospital during the next month by scoring on six statements about the referral process (Fig. 1). A total of 58 GPs volunteered to participate. One form was discarded because of incomplete data.

Each participant was given an identification number. I order to assess external validity we compared the participants with those who did not participate with respect to age, gender, specialty and the scores on the questionnaire. The recorded data were assembled by the first author, who did not see the referral letters, only the referrals registration forms.

### Measurements

In the CPD group meetings the participants scored on ten statements about their usual referring on a 10-cm visual analogue scale (Additional file 1: Appendix 1). During the next month, when actually referring to hospital they used a 10-point Likert scale for the registration of perceived difficulty in referring and patient pressure to be referred, and they marked a priority and wait for the patient, if they had called a hospital specialist when referring and finally the time taken to make the referral. We dichotomized the priority and wait setting into either having marked [1] or not (0) (Additional file 1: Appendix 2). GPs’ gender, age, specialty in family medicine, and location (city or rural) were used to define groups. The number of consultations during the study period was not registered.

### Statistical analyses

For each participant the average score (B1-B6) was calculated as a mean value (B1-2 and B5-6) or a percentage (B3-4) (Table 1). Principal component analysis (PCA) was applied on the 16 variables (A1-10 and B1-6) with oblique rotation (oblimin) which supports improved factor loadings and better interpretability. Bartlett’s test of sphericity was applied to verify if correlations between the variables were sufficiently large for the PCA [21]. The number of principal components retained was based on Kaiser’s criterion of Eigenvalue greater than 1. All extracted components were standardised with mean zero and standard deviation equal to 1. The principal components were used as dependent variables in a multivariate multiple linear regression analysis. The independent variables were GPs’ gender, age, specialty in family medicine and location. To access external validity we compared the questionnaire scores from the participants and non-participants using Student’s unpaired t-test for means [22], Levene’s test of variances, Pearson’s exact chi-square test for proportions and the Wilcoxon-Mann–Whitney test for non-normally distributed variables. A significance level of 0.05 was used for all statistical tests.

IBM SPSS Version 22 was used for all statistical analyses.

### Abduction

The identification and naming of the typologies was done by abduction, a technique described by Umberto Eco in *The sign of three* [23]. Abductive reasoning can be seen as an inference from uncertain data to the possibly best explanation [24]. In this study we used abduction to inference typologies from the components.

## Results

The participants, 58 % males, had a mean age of 49.3, SD (standard deviation): 11.2. 88 % were specialists in family medicine and 70 % worked in urban areas. The participants recorded a total of 691 referrals with a mean value of 12.1 (SD: 5.9) referrals per participating GP. Mean, standard deviation, median and range are presented in Table 1. The mean number of referrals was not significantly different for gender with 11.5 (SD: 4.7) for males and 13.0 (SD: 7.2) for females. The 70 non-participants who only filled in the questionnaire in the CPD group meetings, but did not participate in the recording of data in referrals, had a mean age of 47 years, with 55 % males and 61 % specialists in family medicine. Levene’s test for equality of variances and independent t-test for equality of means showed no significant difference of age between non-participants and participants. Furthermore, the chi square test showed no significant difference for gender between the groups. The proportion of specialists in family medicine was significantly higher (*p* < 0.001) in the participants group. By running Wilcoxon rank-sum tests no significant differences were found between the two populations for the statements A1-10.

### Principal component analysis

The PCA was applied on the 16 variables (A1-10 and B1-6) with oblique rotation (oblimin). Missing values were excluded pairwise given five missing values in A8 and another missing value in A10. Bartlett’s test indicated a sufficient correlation matrix (*p* < 0.001). Using a Kaiser’s criterion of 1, seven components explained 71.1% of the total variance (Table 2). By including component 8 (Eigenvalue: 0.961) 77 % of the total variance could be explained. Table 3 shows the factor loadings after rotation, with loadings over 0.4 highlighted.

### Multivariate multiple linear regression analysis

The multivariate multiple linear regression analysis was performed to investigate the dependency of the eight principal components (PCs) on GPs’ sex, age, specialty in family medicine, location and number of referrals sent. Table 4 shows the eight components and the estimated regression coefficients. One unit increase for a predictor variable leads to an expected change of the PC score equal to the estimated regression coefficient holding all other variables constant. GPs’ gender (*p* = 0.019) and specialty in family medicine (*p* = 0.002) were found to be statistically significant in the combined multivariate test. GPs’ age, location (urban/rural) and the number of referrals recorded were not significant.

The eight principal components describing the different ways GPs think and work when they refer (Table 4) were named:

*1: Fear and uncertainty* (A2, A3, A4). This component describes the fear of having the referral rejected, of not being good enough and not knowing what is expected in a good referral. Non-specialists in family medicine were significantly more insecure than specialists (*p* = 0.015) (Table 4).

*2: Priority decision* (B3, B4)*.* The component identifies GPs who suggested a maximum waiting time and who set a priority for the patient in the referral. Female GPs were making significantly more priority decisions when referring than male GPs (*p* = 0.038).

*3: Completing the referrals during the consultation* (A1, A7)*.* In this component we find GPs scoring low on spending a lot of time and effort on referrals and high on completing the referrals during the consultation.

*4: Little contact with hospital specialist* (B5). High score on this component describes those who seldom contacted a hospital specialist when they referred*.*

*5: Collaboration with patients and colleagues* (A5, A7, A8)*.* This component identifies the GPs who usually complete the referrals during the consultation, who scored high on patients’ participation and opinion being important when they refer and who find it easy to get in contact with a hospital specialist on the phone*.*

*6: Heavy workload* (A6, B1, B6). This component identifies GPs who used more time when they referred, who recorded more difficult referrals and who scored low on the statement that referrals could have been avoided if it was easy to get in contact with a hospital specialist.

*7: Easy support, self-confidence* (A5, A10)*.* This component identifies the GPs who find it easy to get in contact with a hospital specialist and who scored low on the statement that giving the patient a copy of the referral would improve the quality.

*8: Patient pressure, GP reluctance* (A9, B2). In this component we have the GPs who experienced more patient pressure and who indicated reluctance to show the patients the referral or give them a copy. Male GPs scored higher than females (*p* = 0.012) and non-specialists scored higher than specialists in family medicine (*p* = 0.003).

### Two typologies

By abduction [23, 25] of the principal components we found two typologies which describe GPs when they refer:

1. *Confidence* (PC 2, 3, 5) characterizing experienced female GPs who are specialists in family medicine, who involve the patients in the referral process, making priority decisions when they refer, who confer easily with hospital consultants and who complete the referrals during the consultation, without spending too much time.
2. *Uncertainty* (PC 1, 4, 6, 8) characterizing young, male non-specialists in family medicine, expressing fear and uncertainty when they refer, not knowing what is expected in a good referral, with sparse contact with hospital consultants, experiencing heavy workload and pressure from patients to be referred.

## Discussion

Many, mainly male GPs experience heavy work-load and patient pressure when they refer to hospital. We found that a patient-centred way of referring, characterized by easy access to consult a hospital specialist, making priority decisions and completing the referrals during the consultation may be timesaving and associated with less work-load.

### Strengths and limitations

The questionnaire and the referrals registration form were designed by the authors on the basis of the results from a previous study, where we found that many GPs consider referring as asymmetric and sometimes humiliating [13]. The four first statements (A1-4) in the questionnaire focused on problems and uncertainty when referring. Having a special interest in communication in the referral process, GPs’ workload and patients’ pressure to be referred, these are elements which may have had an impact on the choice of questions and statements. Other, more positive and optimistic questions and statements might have given other components and typologies. The questionnaire and the referral registration form were designed in collaboration with experienced academic and non-academic GPs and were piloted among other GPs, without any suggestions for changes. Feedback from the participants supported the assumption that the questions and statements were relevant and easy to score.

The first author was responsible for all information to the participating GPs. Being a colleague and a known person for many of the participants, and having an agenda on a better referral process for all, this personal factor may have a positive impact on the response rate.

The response rate was 44.5 % (19 % of all the GPs in our region) which raises the concern of a selection bias. Similar studies among GPs had response rates from 42-47 % [12, 26]. Among the participants a large part was specialists (88 %) compared with those who didn't participate (61 %). This could affect the interpretation of the results in direction of an over-focus on the *confidence* elements among the experienced specialists, whereas the younger non-specialists over-focused on the *uncertainty* elements may cause a bias which means that the differences between the two typologies are even bigger than in our conclusion. However, as no significant differences were found between participants and non-participants in the 23 CPD group meetings for the statements on the referral process we consider our results to be representative for Norwegian general practice and for countries with similar health care systems.

The questionnaires were filled in anonymously during CPD group meetings, securing each GP’s confidentiality. The participants were instructed to score the referrals consecutively and immediately when or after referring, which is considered to be a strength for the study, because of minimalized recall bias. We have, however no guaranty that all referrals have been registered.

In the PCA, three of our components had two overlapping variables (A5 and 7) (Table 3) meaning components are mainly unique. A 77 % cumulative variance covers most of the variations in the material, indicating an adequate description of the referral process, a considerable strength for our study.

The 57 participants registered a total of 691 referrals during the registration period. As they did not register the number of consultations during this month, we cannot calculate the actual referral rates for our participants, or know if the referral rates were different from those who did not participate. This means that we cannot tell if our participants are within the normal range of variation according to referral rates, or whether this has any impact on the results. Our components and typologies could have been different if we had included the referral rates in the variables for PCA.

By abduction of the eight principal components we found two typologies. Others could have chosen another approach. The principal components are independent quantitative variables, whereas the abductive reasoning can be seen as a creative inference, involving integration and interpretation of ideas to develop new knowledge. In abductive reasoning the premises do not guarantee the conclusion, but can ensure a pragmatic validity [27].

### Comparison with existing literature

This is to our knowledge the first study of typologies of GPs in the referral process. Other studies on typologies in medicine have been done to explore professional identity of nurses [28] and hospital specialists [29]. Our two typologies represent aspects of the referral process where most GPs will recognize themselves. Elements in the *confidence* typology are found in other studies [30, 31]. Collaboration with patients and colleagues are important elements in the referral process, often associated with better health outcomes and improved patient satisfaction [32]. Already in 1992 Huygen et al. found that the integrated style GP can further the health and well-being of their patients [33]. Patients want to know how long they must wait and who they will see [31, 34]. Little et al. found that doctors' behaviour in the consultation was strongly associated with the perceived medical need of the patient, that a minority of examining, prescribing, referrals and investigations were thought by doctors to be slightly needed or not needed at all and that the perceived patient pressure was a strong independent predictor of all doctor behaviours [35]. They concluded: “*To limit unnecessary resource use and iatrogenesis, when management decisions are not thought to be medically needed, doctors need to directly ask patients about their expectations”.* Ringberg et al. found that the issue of the referral was introduced by the patients in 29.4 % of cases [11]. Our finding echoes these results and the results of Donohoe et al., who found that patients’ requests influenced referral decisions in one fifth of the cases [36]. Ringberg et al. found that female GPs referred more often than male to reassure the patient because they experienced lack of medical knowledge and when the issue of referring was introduced [12]. A low referral rate was one of the characteristics of the integrated practice style, with maximum scores on patient- and goal-oriented approaches. Low referrers were more confident about their decisions, more positive about alternatives to hospital admission and more able to resist pressure from families and carers to have someone admitted; they saw hospitals as places to be avoided and viewed their goal as preventing an admission [10].

The *uncertainty* typology matches our findings in a previous study, where we found that many GPs consider referring to be asymmetric and sometimes embarrassing [13]. Other studies have shown that younger doctors are more vulnerable to patients’ scepticism and criticism, and that individual uncertainty among GPs about referring has a significant impact on higher referral rates [10–12, 16, 37]. Calnan et al. found that high-referring GPs were more cautious and believed that it was better to admit if in doubt. The high referrers in their study expressed anxiety about the consequences of a decision not to admit, both for the patient and for themselves and they held negative attitudes towards alternatives to hospital admission. The *uncertainty* typology encompasses those who seldom contacted a hospital specialist when they referred*.* In Berendsen et al’s study 73.2 % of GPs answered that a hospital specialist could easily be reached for a colleague consultation [26]. Earlier studies have shown that both GPs and hospital consultants called for more contact and communication in the referral process [13, 14]. *Heavy workload* describes a well-known situation for many GPs, who use much time when they refer, experiencing many difficult referrals and who do not think that referrals could have been avoided if they called a hospital specialist. In an Israeli study published in 2014 Kushnir et al. found higher referral rates for diagnostic tests and specialist clinics for physicians with burnout symptoms and when objective workload increased [38].

The last years’ development of better e-communication and more advanced electronic referral decision support systems have facilitated an easier referral process [18, 19], making it more convenient to complete the referrals during the consultation, which may be timesaving and associated with less work-load.

Our results support the conclusion in Calnan et al’s study, which calls for educational programmes to improve GPs’ judgements of their competences and to build appropriate levels of confidence [10]. Our study adds that a patient-centred practice, easy access to confer with a hospital consultant and good cooperation with patients when making the referrals may be a major topic for CPD groups and vocational training for specialists in family medicine.

## Conclusions

Training collaboration with patients and hospital consultants may foster both self-reflections on own competences and increased levels of confidence when referring. Our results need further research to investigate the impact on the quality of the referral process and the consequences for patients and their clinical pathways.

## Additional file

Additional file 1: Appendix 1. Questions about the referral process to hospital for non-urgent patients. **Appendix 2.** Referral registration form. (DOCX 16 kb)

## References

[1] Loudon I (2008). The principle of referral: the gatekeeping role of the GP. Br J Gen Pract.

[2] Barnett ML, Song ZLandon BE (2012). Trends in physician referrals in the United States, 1999–2009. Arch Intern Med.

[3] O'Donnell CA (2000). Variation in GP referral rates: what can we learn from the literature?. Fam Pract.

[4] Starfield B, Shi LMacinko J (2005). Contribution of primary care to health systems and health. Milbank Q.

[5] McBride D, Hardoon S, Walters K (2010). Explaining variation in referral from primary to secondary care: cohort study. BMJ.

[6] van Dijk CE, Korevaar JC, Koopmans B (2014). The primary-secondary care interface: Does provision of more services in primary care reduce referrals to medical specialists?. Health Policy.

[7] Godager G, Iversen TMa CT (2015). Competition, gatekeeping, and health care access. J Health Econ.

[8] Forrest CB, Nutting PA, Starfield B (2002). Family physicians' referral decisions: results from the ASPN referral study. J Fam Pract.

[9] Forrest CB, Nutting PA, von Schrader S (2006). Primary care physician specialty referral decision making: patient, physician, and health care system determinants. Med Decis Making.

[10] Calnan M, Payne S, Kemple T (2007). A qualitative study exploring variations in GPs' out-of-hours referrals to hospital. Br J Gen Pract.

[11] Ringberg U, Fleten N, Deraas TS (2013). High referral rates to secondary care by general practitioners in Norway are associated with GPs' gender and specialist qualifications in family medicine, a study of 4350 consultations. BMC Health Serv Res..

[12] Ringberg U, Fleten NForde OH (2014). Examining the variation in GPs' referral practice: a cross-sectional study of GPs' reasons for referral. Br J Gen Pract.

[13] Thorsen O, Hartveit MBaerheim A (2012). General practitioners' reflections on referring: an asymmetric or non-dialogical process?. Scand J Prim Health Care.

[14] Thorsen O, Hartveit MBaerheim A (2013). The consultants' role in the referring process with general practitioners: partners or adjudicators? a qualitative study. BMC Fam Pract.

[15] Xiang A, Smith H, Hine P (2013). Impact of a referral management "gateway" on the quality of referral letters; a retrospective time series cross sectional review. BMC Health Serv Res.

[16] Espeland ABaerheim A (2007). General practitioners' views on radiology reports of plain radiography for back pain. Scand J Prim Health Care.

[17] Akbari A, Mayhew A, Al-Alawi MA (2008). Interventions to improve outpatient referrals from primary care to secondary care. Cochrane Database Syst Rev.

[18] Rokstad IS, Rokstad KS, Holmen S (2013). Electronic optional guidelines as a tool to improve the process of referring patients to specialized care: An intervention study. Scand J Prim Health Care.

[19] Mariotti G, Gentilini MDapor V (2013). Improving referral activity on primary-secondary care interface using an electronic decision support system. Int J Med Inform.

[20] Målbeskrivelse og gjennomføringsplan for allmennmedisin Tidsskr Nor Legefor2013. Available from: http://legeforeningen.no/PageFiles/540/Allmenn_2013_gyldig.pdf.

[21] Field A (2000). Discovering statistics using SPSS for Windows: advanced techniques for the beginner.

[22] Student. The probable error of mean. Biometrika. 1908;VI(1):1–25.

[23] Eco U. Horns H, Insteps. In Eco U, Sebeok TA (eds.). Dupin, Holmes, Peirce. The sign of three. Dupin, Holmes, Peirce. USA: Indiana University Press; 1988.

[24] Lawson AE, Daniel ES (2011). Inferences of clinical diagnostic reasoning and diagnostic error. J Biomed Inform.

[25] Mirza NA, Akhtar-Danesh N, Noesgaard C (2014). A concept analysis of abductive reasoning. J Adv Nurs.

[26] Berendsen AJ, Kuiken A, Benneker WH (2009). How do general practitioners and specialists value their mutual communication? A survey. BMC Health Serv Res.

[27] Kvale S (1996). Interviews : an introduction to qualitative research interviewing.

[28] Hensel D (2014). Typologies of professional identity among graduating baccalaureate-prepared nurses. J Nurs Scholarsh.

[29] Forrest CB (2009). A typology of specialists' clinical roles. Arch Intern Med.

[30] Shin DW, Roter DL, Roh YK (2015). Physician gender and patient centered communication: the moderating effect of psychosocial and biomedical case characteristics. Patient Educ Couns.

[31] Cox K, Britten N, Hooper R (2007). Patients' involvement in decisions about medicines: GPs' perceptions of their preferences. Br J Gen Pract.

[32] Raina RS, Singh P, Chaturvedi A (2014). Emerging ethical perspective in physician-patient relationship. J Clin Diagn Res.

[33] Huygen FJ, Mokkink HG, Smits AJ (1992). Relationship between the working styles of general practitioners and the health status of their patients. Br J Gen Pract.

[34] Banks J, Walter FM, Hall N (2014). Decision making and referral from primary care for possible lung and colorectal cancer: a qualitative study of patients' experiences. Br J Gen Pract.

[35] Little P, Dorward M, Warner G (2004). Importance of patient pressure and perceived pressure and perceived medical need for investigations, referral, and prescribing in primary care: nested observational study. BMJ.

[36] Donohoe MT, Kravitz RL, Wheeler DB (1999). Reasons for outpatient referrals from generalists to specialists. J Gen Intern Med.

[37] Ghosh AK (2004). Understanding medical uncertainty: a primer for physicians. J Assoc Physicians India.

[38] Kushnir T, Greenberg D, Madjar N (2014). Is burnout associated with referral rates among primary care physicians in community clinics?. Fam Pract.

