# Erratum to: Typologies in GPs’ referral practice

**DOI:** 10.1186/s12875-016-0572-2

**Published:** 2017-01-31

**Authors:** Olav Thorsen, Miriam Hartveit, Jan Olav Johannessen, Lars Fosse, Geir Egil Eide, Jörn Schulz, Anders Bærheim

**Affiliations:** 1 Department of Global Public Health and Primary Care, University of Bergen, Box 7800, Bergen, N-5020 Norway; 2 Department of Research, Stavanger University Hospital, Box 8100, Stavanger, N-4068 Norway; 3 Department of Orthopaedics, Stavanger University Hospital, Box 8100, Stavanger, N-4068 Norway; 4 Centre for Clinical Psychosis Research, Division of Psychiatry, Stavanger University Hospital, Box 8100, Stavanger, N-4068 Norway; 5 Faculty of Social Sciences, University of Stavanger, Box 8100, Stavanger, N-4068 Norway; 6 Section for Research and Innovation, Helse Fonna HF, Box 2170, Haugesund, N-5504 Norway; 7 Centre for Clinical Research, Haukeland University Hospital, Box 1400, Bergen, N-5021 Norway; 8 Section of Biostatistics, Stavanger University Hospital, Box 8100, Stavanger, N-4068 Norway

## Erratum

The original article contained a major omission whereby Tables 1, 2, 3, 4 were mistakenly left out from the article body; this error was carried forward by the production team handling this article, and thus was not the fault of the authors.

As such, the original article has now been updated to include these tables.

**Table 1:** Norwegian general practitioners’ scores on statements about their referral process (A1-10) and data collected when actually referring to hospital (B1-6) during 1 month in 2014 (*n* = 57)

Variables		Mean	SD	Median	Min	Max
Statements on VAS 10 cm: 0 = strongly disagree, 10 = strongly agree)						
A1. “I spend a lot of time and effort on referrals”		5.3	2.0	5.2	0.5	9.8
A2. “I often feel that I don’t know enough about what is expected to make a good referral”		3.2	2.1	2.5	0.0	10.0
A3. “I am often afraid to have the referral rejected from hospital”		1.4	1.5	1.0	0.0	8.0
A4. “I am often afraid that the referral gives an impression of me not knowing enough about the actual medical problem”		2.9	2.2	2.0	0.0	9.5
A5. “It is easy to get in contact with a hospital specialist for advice”		4.9	2.3	5.0	1.0	9.0
A6. “Some referrals could have been avoided if I had got in contact with a hospital consultant when referring”		5.8	3.0	6.5	0.0	10.0
A7. “I usually complete the referral during the consultation”		4.6	3.3	5.0	0.0	10.0
A8. “The patient’s participation and opinion is important to me when I refer”		6.2	1.9	6.3	2.0	9.5
A9. “The patient should see the referral or have a copy before it is sent”		5.0	2.8	5.0	0.3	10.0
A10 “Giving the patient a copy of the referral will improve the quality”		4.4	2.8	5.0	0.5	10.0
B1. Difficult referral to make	(Likert scale 1–10)	2.6	1.0	2.7	1.0	5.6
B2. Pressure from patient to be referred	(Likert scale 1–10)	2.0	0.8	1.0	1.0	4.7
B3. Suggesting a priority for the patient to be admitted to hospital (%)		39.9	39.3	26.0	0.0	100.0
B4. Suggesting a wait for the patient to be admitted to hospital (%)		28.2	33.6	17.6	0.0	100.0
B5. Telephone contact with hospital specialist when referring (%)		9.1	16.1	0.0	0.0	100.0
B6. The time used for making the referral (minutes)		8.2	3.5	7.5	2.0	17.1

*Abbreviations:* GP: General practitioner; SD: standard deviation; VAS: visual analogue scale; Min: minimum, Max: maximum

**Table 2:** Eigenvalues and cumulative variance of the first ten components in a principal component analysis of 16 variables of the referral process from 57 general practitioners in Norway during spring 2014

Initial eigenvalues			
Component	Total	% of variance	Cumulative %
1	2.3	14.4	14.4
2	1.9	12.0	26.5
3	1.7	10.9	37.3
4	1.6	10.0	47.3
5	1.4	8.5	55.8
6	1.3	8.3	64.1
7	1.1	7.0	71.1
8	1.0^a^	6.0	77.1
9	0.9	5.3	82.4
10	0.8	5.1	87.5

^a)^0.961

**Table 3:** Rotated pattern matrix after principal component analysis^a)^ of 16 variables of the referral process from 57 general practitioners in Norway during spring 2014

Components								
Variables	1	2	3	4	5	6	7	8
A3: Afraid of rejection of referral	**.872**	.052	**-**.056	.031	**-**.051	.124	.038	**-.**040
A4: Not being good enough	**.864**	**-**.131	**-**.114	**-**.066	**-**.055	.021	**-**.176	.020
A2: Unknown expectations	**.661**	**-**.050	.246	.015	.060	**-**.130	.383	**-**.044
B4: Suggested waiting	**-**.029	**.826**	.252	.150	**-**.264	**-**.066	**-**.074	**-**.071
B3: Priority in referral	**-**.159	**.760**	**-**.152	.028	.370	.157	.056	.030
A1: Using much time to refer	.043	**-**.148	-**.910**	.110	.108	.021	**-**.039	**-**.123
A7: Referral in consultation	**-**.013	**-**.138	**.690**	.062	**.407**	.111	**-**.068	**-**.187
B5: Conferred with consultant	.026	**-**.127	.103	-**.950**	.056	.097	**-**.078	.147
A8: Patient opinion important	**-**.068	.002	.085	**-**.040	**.841**	**-**.037	**-**.108	**-**.196
A5: Contact with consultant	**-**.023	.021	**-**.139	.080	**.431**	.041	**.431**	.373
B6: Time used to refer	.043	.027	**-**.025	**-**.346	.027	**.848**	.124	**-**.095
B1: Difficult referral	.152	.091	.083	.351	.006	**.713**	**-**.287	.279
A6: Referral avoided if contact	.308	.373	**-**.100	**-**.048	.333	-**.426**	**-**.240	.145
A10: Copy gives better quality	**-**.020	.020	**-**.009	**-**.027	.118	**-**.017	-**.873**	.038
A9: Referral copy to patient	.033	**-**.060	.036	.247	.213	**-**.022	**-**.007	-**.795**
B2: Patient pressure	**-**.004	**-**.343	.198	.356	.084	.004	**-**.095	**.601**

^a)^Using an oblique (oblimin) rotation with Kaiser normalisation. Loadings larger than 0.4 are highlighted

**Table 4:** Results from multivariate multiple linear regression analysis of eight principal components on referrals from 57 general practitioners (GPs) in Norway in 2014

Dependent variables: Typological components									
Independent variables	1	2	3	4	5	6	7	8	Multivariate
	b (*p*-value)	b (*p*-value)	b (*p*-value)	b (*p*-value)	b (*p*-value)	b (*p*-value)	b (*p*-value)	b (*p*-value)	*p*-value
GP age	**−**0.01 (0.469)	0.01 (0.780)	0.01 (0.727)	0.01 (0.904)	0.01 (0.594)	0.01 (0.580)	0.02 (0.235)	−0.01 (0.791)	.965
Gender: male	−0.23 (0.412)	**−0.63 (0.038)**	0.54 (0.068)	−0.22 (0.463)	0.07 (0.815)	0.57 (0.069)	0.34 (0.254)	**0.69 (0.012)**	**.019**
Specialty: no	**1.32 (0.015)**	−0.13 (0.822)	0.79 (0.148)	0.16 (0.770)	0.08 (0.892)	0.84 (0.146)	0.83 (0.145)	**1.52 (0.003)**	**.002**
Location: urban	−0.39 (0.214)	−0.12 (0.714)	−0.16 (0.624)	0.48 (0.157)	−0.51 (0.138)	−0.45 (0.189)	−0.06 (0.860)	−0.12 (0.684)	.269
N referrals	−0.01 (0.893)	0.02 (0.346)	0.04 (0.090)	0.05 (0.049)	0.01 (0.575)	0.02 (0.519)	−0.03 (0.258)	0.05 (0.020)	.056

b: Estimated regression coefficients; *p*-values from t-test
