# Supplementary material for: Typologies in GPs’ referral practice
Source: BMC Fam Pract. 2016 Jul 18;17:76. doi: 10.1186/s12875-016-0495-y (PMC4949760; doi:10.1186/s12875-016-0495-y)
Supplement: Additional file 1: Appendix 1. — Questions about the referral process to hospital for non-urgent patients. Appendix 2. Referral registration form. (DOCX 16 kb) [file 12875_2016_495_MOESM1_ESM.docx]

**Appendix 1**

**Questions about the referral process to hospital for non-urgent patients**

**First some information about you:**

Man: |_|

Woman: |_| Age: |__|__| Specialist in general practice? Yes |_| No |_|

**Mark on the line how much you agree on these statements:**

1. **I spend a lot of time and effort on making the referrals**

Disagree |---------------------------------------------------------------------------| Totally agree

1. **I often feel that I don’t know enough about what is expected to make a good referral**

Disagree |---------------------------------------------------------------------------| Totally agree

1. **I am often afraid to have the referral rejected from hospital**

Disagree |---------------------------------------------------------------------------| Totally agree

1. **I am often afraid that the referral gives an impression of me not knowing enough about the actual medical problem**

Disagree |---------------------------------------------------------------------------| Totally agree

1. **It is easy to get in contact with a hospital consultant for an advice**

Disagree |---------------------------------------------------------------------------| Totally agree

1. **Some referrals could have been avoided if I had got in contact with a hospital consultant when referring**

Disagree |---------------------------------------------------------------------------| Totally agree

1. **I usually complete the referral during the consultation**

Disagree |---------------------------------------------------------------------------| Totally agree

1. **Patient’s participation and opinion is important to me when I refer**

Disagree |---------------------------------------------------------------------------| Totally agree

1. **The patient should see the referral or have a copy before it is sent**

Disagree |---------------------------------------------------------------------------| Totally agree

1. **Giving the patient a copy of the referral will improve the quality of the referral**

Disagree |---------------------------------------------------------------------------| Totally agree

***Thank you for your cooperation!***

**Appendix 2**

**Referral registration form**

| **Date** | **Patient’s birth year:** | **Patient’s**  **gender:**  1=male  2=female | **Easy or difficult referral to make?** (1-10)  1= very easy  10=very difficult | **Did you feel pressured by the patient to be referred?**  **(1-10)**  1=not at all!  10= yes absolutely! | **My suggestion for priority**  (1-3) according to national guidelines | **My suggestion for wait**  (weeks) | **Did you call a hospital specialist when referring?**  1 = Yes,  2 = No  3 = I tried, but no contact | **How long time**  (minutes) **did it take to make this referral?** |
| --- | --- | --- | --- | --- | --- | --- | --- | --- |
|  |  |  |  |  |  |  |  |  |
|  |  |  |  |  |  |  |  |  |
